# Variation in public health reimbursement policies of ECT and rTMS across 28 European countries: A call for evidence-based policy reform from the ECNP Neuromodulation Network

**DOI:** 10.1016/j.nsa.2026.107030

**Published:** 2026-09-23

**Authors:** Nils Opel, Jovana Bjekić, Jerome Brunelin, Jean-Marie Batail, Annemiek Dols, Sara Assecondi, Sharmili Edwin Thanarajah, Leif Oltedal, Tiago Costa, Piotr Jażdżyk, Axel Nordenskjöld, Katya Rubia, Monika Klírová, Debby Klooster, Astrid Kibleur, Ana Ganho-Ávila, Odile A. van den Heuvel, Jonathan Repple

**Affiliations:** a Department of Psychiatry and Neuroscience, Campus Benjamin Franklin, Charité–Universitätsmedizin Berlin, Berlin, Germany; b German Center for Mental Health (DZPG), Germany; c Human Neuroscience Group, Center for Neuroscience and Neuromodulation, Institute for Medical Research, University of Belgrade, Belgrade, Serbia; d Université Claude Bernard Lyon 1, CNRS, INSERM, Centre de Recherche en Neurosciences de Lyon (CRNL), Bron, France; e Pôle Hospitalo-Universitaire de Psychiatrie Adulte, Centre Hospitalier Guillaume Régnier, Rennes, France; f Univ Rennes, Inria, CNRS, Inserm, IRISA UMR 6074, Empenn ERL U1228, Rennes, France; g Centre d’Investigation Clinique de Rennes (CIC 1414 Inserm), Rennes, France; h Université de Rennes, Rennes, France; i Department of Psychiatry, UMC Utrecht Brain Center, University Medical Center Utrecht, Utrecht University, Utrecht, the Netherlands; j Psychology and Cognitive Science - DiPSCo, University of Trento, Rovereto, Italy; k Department of Psychiatry, Psychosomatic Medicine and Psychotherapy, University Hospital Frankfurt, Goethe University Frankfurt, Frankfurt, Germany; l Cooperative Brain Imaging Center (CoBIC), Goethe University Frankfurt, Frankfurt, Germany; m Department of Clinical Medicine, University of Bergen, Bergen, Norway; n Mohn Medical Imaging and Visualization Centre, Department of Radiology, Haukeland University Hospital, Bergen, Norway; o Translational and Clinical Research Institute, Newcastle University, Newcastle upon Tyne, United Kingdom; p Cumbria, Northumberland, Tyne and Wear NHS Foundation Trust, Newcastle upon Tyne, United Kingdom; q NIHR Newcastle Biomedical Research Centre, Newcastle upon Tyne, United Kingdom; r Department of Psychiatry, Medical University of Warsaw, Warsaw, Poland; s University Health Care Research Centre, Faculty of Medicine and Health, Örebro University, Örebro, Sweden; t Department of Child and Adolescent Psychiatry, Institute of Psychiatry, Psychology and Neuroscience, King's College London, London, United Kingdom; u Mental Health Dresden–Leipzig, Technical University Dresden, Dresden, Germany; v National Institute of Mental Health, Klecany, Czech Republic; w Third Faculty of Medicine, Charles University, Prague, Czech Republic; x Electromagnetics for Care and Cure Laboratory, Department of Electrical Engineering, Eindhoven University of Technology, Eindhoven, the Netherlands; y Department of Clinical Physics, Epilepsy Center Kempenhaeghe, Heeze, the Netherlands; z Department of Nuclear Medicine, CHU de Caen Normandie, Normandie Université, UNICAEN, Caen, France; aa LIP/PC2S, Université Savoie Mont Blanc, Université Grenoble Alpes, Chambéry, France; ab Centre for Research in Neuropsychology and Cognitive Behavioural Interventions, Faculty of Psychology and Educational Sciences, University of Coimbra, Coimbra, Portugal; ac Faculty of Psychology and Educational Sciences, University of Coimbra, Coimbra, Portugal; ad Department of Psychiatry and Department of Anatomy, Amsterdam University Medical Center, Vrije Universiteit Amsterdam, Amsterdam, the Netherlands; ae Amsterdam Neuroscience, Compulsivity, Impulsivity and Attention Program, Amsterdam, the Netherlands; af Institute for Translational Psychiatry, University of Münster, Münster, Germany; ag Department of Cognitive Neuroscience, Faculty of Psychology, Maastricht University, Maastricht, 6201 BC, the Netherlands; ah Goethe University Frankfurt, Cooperative Brain Imaging Center - CoBIC, Frankfurt, Germany

## Abstract

Mental disorders contribute significantly to the disease burden in Europe. Brain stimulation treatments such as electroconvulsive therapy (ECT) and repetitive transcranial magnetic stimulation (rTMS) are supported by strong evidence and are recommended in clinical guidelines in many countries. However, access to these interventions remains highly inconsistent across Europe.

Reimbursement by public health services plays a critical role in enabling equitable access to these therapies. In this study, we examined disparities in national reimbursement policies for ECT and rTMS for the treatment of mental disorders in both inpatient and outpatient settings across 28 European countries, leveraging information from public databases and expertise from a multinational expert group within the ECNP Neuromodulation Network.

Our results show that ECT is reimbursed for inpatient care in all 28 studied countries, while outpatient ECT is reimbursed in 15 of 28 countries (53.6%), with a geographic trend toward higher availability in Scandinavian and Western European countries. rTMS reimbursement was reported in 15 of 28 countries (53.6%) for inpatient care and in 8 of 28 countries (28.6%) for outpatient care, with no consistent geographic pattern. Subsequent analyses examining reimbursement policies in relation to the national Human Development Index (HDI), the Gini coefficient, and healthcare infrastructure metrics revealed no statistically significant differences between countries that do and do not reimburse outpatient ECT. Although countries reimbursing outpatient ECT showed numerically fewer hospital beds and slightly higher HDI values, these differences did not reach statistical significance. Similarly, no significant associations were observed between rTMS reimbursement and indicators of socioeconomic development, income inequality, or healthcare infrastructure.

These findings highlight a striking disparity in national reimbursement policies across Europe, despite the solid evidence base for ECT and rTMS in treating mental health disorders. The current landscape suggests that many patients in Europe experience limited access to these effective treatments that could alleviate individual suffering and reduce societal disease burden. We call for urgent policy action to align health system reimbursement with clinical evidence for treatment efficacy and to improve access to evidence-based brain stimulation therapies across Europe.

## 1 Introduction

Mental disorders are a leading cause of disease burden in Europe (Efficacy and safety of electroconvulsive, 2003; Vida et al., 2023). This is mainly due to their high prevalence and, more importantly, their often recurrent and chronic course, which can severely impair individuals’ daily functioning and quality of life. The typical first-line treatments for depressive disorders, such as antidepressant medications and psychotherapy, have modest response rates, with approximately 30–40% of patients not responding to initial treatment (Ramos et al., 2025; Semple et al., 2024). This leaves a significant number of patients with residual symptoms or ongoing illness, contributing to substantial personal, social, and economic costs (Bulteau et al., 2022).

Non-invasive brain stimulation techniques, most notably electroconvulsive therapy (ECT) (Efficacy and safety of electroconvulsive, 2003) and repetitive transcranial magnetic stimulation (rTMS) (Vida et al., 2023; Ramos et al., 2025) represent evidence-based alternative therapeutic options for a wide range of mental disorders. Over decades of research, both modalities have demonstrated robust efficacy in patients who do not respond to standard interventions, with reported response rates in depression of approximately 60–80% for ECT (Efficacy and safety of electroconvulsive, 2003; Semple et al., 2024) and 30–60% for rTMS (Bulteau et al., 2022; Trapp et al., 2025) depending on the population and study design. While ECT for depression is generally associated with higher efficacy, it also carries a greater burden of cognitive side effects and logistical constraints. In contrast, rTMS offers a more favorable side-effect profile with reduced overall burden for patients (Trapp et al., 2025; Chen et al., 2025).

Due to the longstanding and strong body of evidence supporting their efficacy and safety, both ECT and rTMS are now included in many national and international treatment guidelines for affective disorders (Hussain et al., 2024; Milev et al., 2016; AWMF Leitlinienregister; Depression in adults, 2022; Ministarstvo et al.; Lefaucheur et al., 2020; Hebel et al., 2022). However, despite these clear recommendations, the implementation and availability of these treatments still vary widely across European countries. Among the various barriers likely contributing to this discrepancy, such as logistical challenges, limited access to equipment, and shortages of trained professionals, differences in public health reimbursement policies may represent the most critical limiting factor.

In most European countries, public health services are expected to provide coverage for evidence-based treatments. When such treatments are not reimbursed, despite inclusion in guidelines and demonstrated efficacy, mental health care institutions may choose not to provide them and clinicians and patients may struggle to access them. This may create substantial inequities, especially for those who cannot afford out-of-pocket payment, thereby further widening existing treatment gaps in mental health care. Moreover, lack of reimbursement can inadvertently signal uncertainty about the treatment's legitimacy or safety, potentially discouraging both prescribers and patients from pursuing its use.

Understanding and addressing these reimbursement disparities is therefore essential not only for improving access to evidence-based care but also for promoting equity in mental health service provision across Europe. Systematic alignment of reimbursement policies with clinical evidence could be a key lever in improving outcomes for individuals with mental disorders.

In this study, we investigated the variation in current public reimbursement policies for ECT and rTMS for the treatment of mental disorders across European countries, examining both inpatient and outpatient settings. Our analysis was based on publicly available data sources and expert input from a cross-national network of mental health professionals within the European College of Neuropsychopharmacology (ECNP), i.e., the ECNP Neuromodulation Network (https://www.ecnp.eu/research/ecnp-networks/list-of-ecnp-networks/neuromodulation-network/).

## 2 Methods

### 2.1 Databases & search strategy

We conducted a systematic search of publicly available databases in 28 European countries to assess reimbursement policies for both electroconvulsive therapy (ECT) and repetitive transcranial magnetic stimulation (rTMS) for any mental disorder, independently examining their status in inpatient and outpatient treatment settings. Each search was performed by at least two independent researchers and cross-checked for consistency. The primary sources of information were official public databases maintained by national governments or healthcare-related institutions. Reimbursement information was collected between October and December 2025 and reflects policies in place during this period. Searches were performed using standardized terms related to ECT, rTMS, reimbursement, insurance coverage, and public health services, adapted where necessary to the local language. In some cases, supplementary sources, such as peer-reviewed publications, policy reports, or direct communication with local experts, were also consulted to confirm or complement the findings; local experts were consulted particularly when reimbursement information from public databases was unclear or incomplete. All data entries were only included if the information could be independently verified by a second researcher. Detailed references for each country's data sources are provided in Supplementary Table 1.

For all countries included in the final analysis, we incorporated key indicators of national development and healthcare infrastructure to examine whether these factors systematically varied with reimbursement policies. Specifically, we collected data on the Human Development Index (HDI) (Country-insights; Nordanskog et al., 2015; Wilhelmy et al., 2020; Jolivet and Grözinger, 2021), a composite measure of life expectancy, education, and income, the Gini coefficient (SI.POV.GINI.), an indicator of income inequality within a population, population density (EN.POP.DNST.), the number of physicians per 1,000 inhabitants (SH.MED.PHYS.ZS.), and the number of hospital beds per 1,000 inhabitants (SH.MED.BEDS.ZS.).

### 2.2 Statistical analysis

To examine whether reimbursement status was associated with national development and healthcare infrastructure indicators, countries were grouped according to reimbursement status (yes/no) separately for outpatient ECT and for rTMS (any setting), and each of the five indicators (HDI, Gini coefficient, population density, physicians per 1,000 inhabitants, and hospital beds per 1,000 inhabitants) was compared between groups. Given the small sample size and the non-normal distribution of several indicators (Shapiro-Wilk test), group differences were assessed using two-sided Mann-Whitney U tests. A significance threshold of p < 0.05 was applied. All statistical analyses were performed using Python (Python Software Foundation, Wilmington, DE, USA) with the SciPy ecosystem, including NumPy, pandas, SciPy, and statsmodels.

### 2.3 Exclusion and inclusion criteria

We screened publicly available sources on reimbursement policies of national public health insurance providers for ECT and rTMS across all 44 countries in the broader European region. The only inclusion criterion was geographic location within this region. Private insurance coverage was not considered; analyses focused solely on reimbursement by national public health insurance/provider systems. Reimbursement was deemed available if nationwide policies for the respective treatment for any mental disorder were identified. Countries were excluded if reliable data on reimbursement for either ECT or rTMS could not be obtained from public databases or verified through supplementary sources. When reimbursement information was unclear or incomplete, local experts were consulted, and information was only retained if independently verified by a second researcher. Based on this criterion, 16 countries were excluded due to insufficient or unavailable information, resulting in a final sample of 28 countries included in the full analysis (see Supplementary Table 1).

### 2.4 Included countries

*W*e systematically analyzed reimbursement policies for ECT and rTMS across 28 European countries representing diverse geographic regions, including Scandinavia, the British Isles, Central, Eastern, and Southern Europe. Countries were included based on the availability of online resources or expert knowledge, with efforts made to ensure broad regional representation and substantial coverage of the overall European population. The countries investigated were: Austria, Belgium, Bosnia and Herzegovina, Croatia, Czech Republic, Denmark, Finland, France, Germany, Greece, Ireland, Israel, Italy, Lithuania, Luxembourg, the Netherlands, Norway, Poland, Portugal, Romania, Serbia, Slovakia, Sweden, Switzerland, Turkey, Spain, Hungary and the United Kingdom (UK). Collectively, these countries represent an estimated population of approximately 670 million inhabitants, accounting for ∼83% of the total European population, and covering 28 out of the 44 countries typically considered part of the broader European region.

### 2.5 Involved researchers

This study was conducted as part of the ECNP Neuromodulation Network, with all core network members actively involved in the search, verification, and interpretation of reimbursement data. Consequently, the research team comprised experts from Belgium, the Czech Republic, Denmark, France, Germany, Italy, the Netherlands, Norway, Poland, Portugal, Serbia, Sweden, and the UK, ensuring broad geographic representation. This collaborative, cross-national approach was intentionally chosen to leverage local expertise and first-hand knowledge of clinical practices related to brain stimulation techniques, thereby enhancing the validity and contextual relevance of the findings across diverse public healthcare systems in Europe.

## 3 Results

### 3.1 ECT

We found that inpatient ECT was reimbursed in all countries included in the final analysis. In contrast, outpatient ECT was reimbursed in only 15 out of 28 countries (53.6%). A notable geographic pattern emerged: all Scandinavian countries, along with several Central and Western European nations, including Belgium, the Netherlands and France, offered reimbursement for outpatient ECT (Fig. 1). In Germany, however, although outpatient ECT is theoretically reimbursable, the existing reimbursement framework fails to provide sufficient financial support for service provision and as a result, outpatient ECT is not performed in clinical practice. Among Eastern European countries, outpatient ECT reimbursement was generally lacking, with the exception of Czech Republic.

**Fig. 1:**
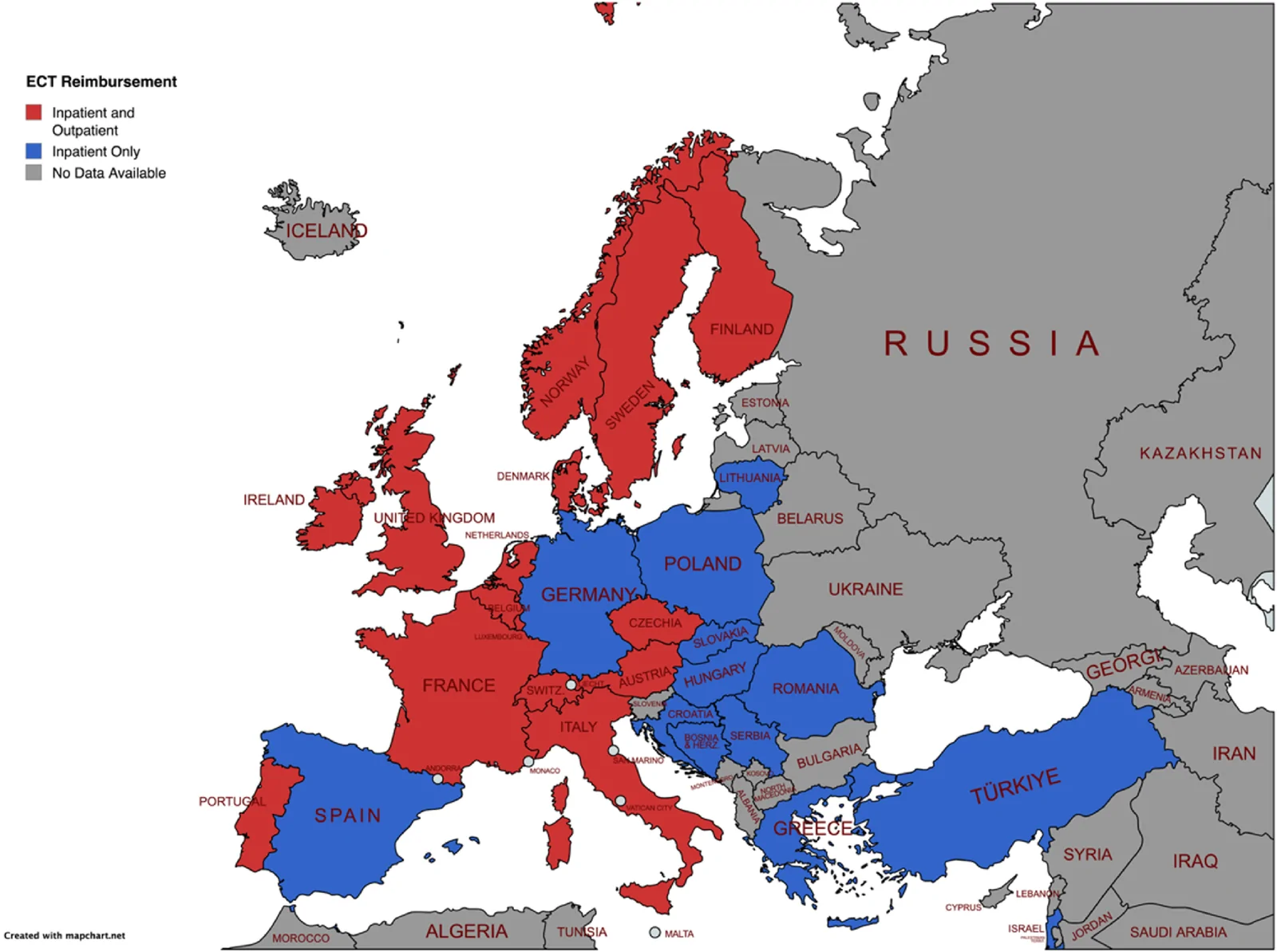
Visualisation of reimbursement status for ECT in inpatient and outpatient settings across Europe.

### 3.2 rTMS

Reimbursement for rTMS (in either inpatient or outpatient settings) was available in 15 of 28 countries (53.6%). Specifically, rTMS reimbursement was available for 15/28 (53.6%) countries for inpatient care and in 8/28 (28.6%) for outpatient care. In contrast to ECT, no clear geographic pattern emerged in the distribution of rTMS reimbursement policies across the included countries (Fig. 2).

**Fig. 2:**
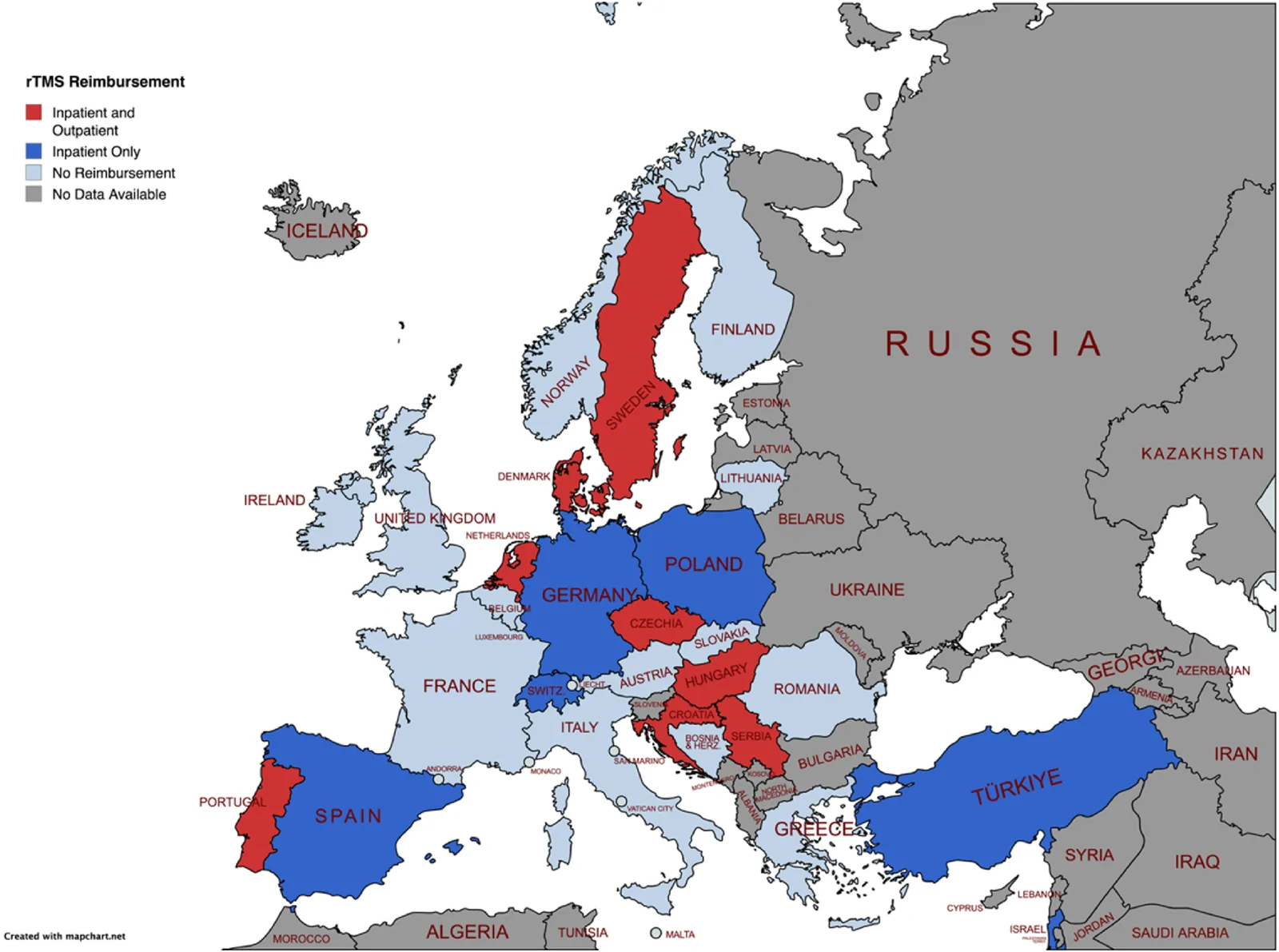
Visualisation of reimbursement status for rTMS in inpatient and outpatient settings across Europe.

### 3.3 Variation by development indices

In addition to geographic patterns, we examined potential associations between reimbursement status and national development indicators, including the HDI, the Gini coefficient, population density, and healthcare infrastructure metrics such as the number of physicians and hospital beds per capita, using Mann-Whitney U tests (see Statistical analysis, Methods; Supplementary Table 2). No significant associations emerged between reimbursement status and national development indicators, either for outpatient ECT or for rTMS reimbursement. Countries reimbursing outpatient ECT showed numerically different values for hospital beds per 1,000 inhabitants (median = 3.1, SD = 1.7 vs. median = 4.2, SD = 1.7, p = 0.153) and HDI (median = 0.930, SD = 0.047 vs. median = 0.915, SD = 0.041, p = 0.447), but neither difference reached statistical significance (Fig. 3, Supplementary Table 2). Similarly, no significant differences in HDI, Gini coefficient, population density, physicians per 1,000 inhabitants, or hospital beds per 1,000 inhabitants were observed between countries with and without rTMS reimbursement (all p > 0.05; Fig. 4, Supplementary Table 2).

**Fig. 3:**
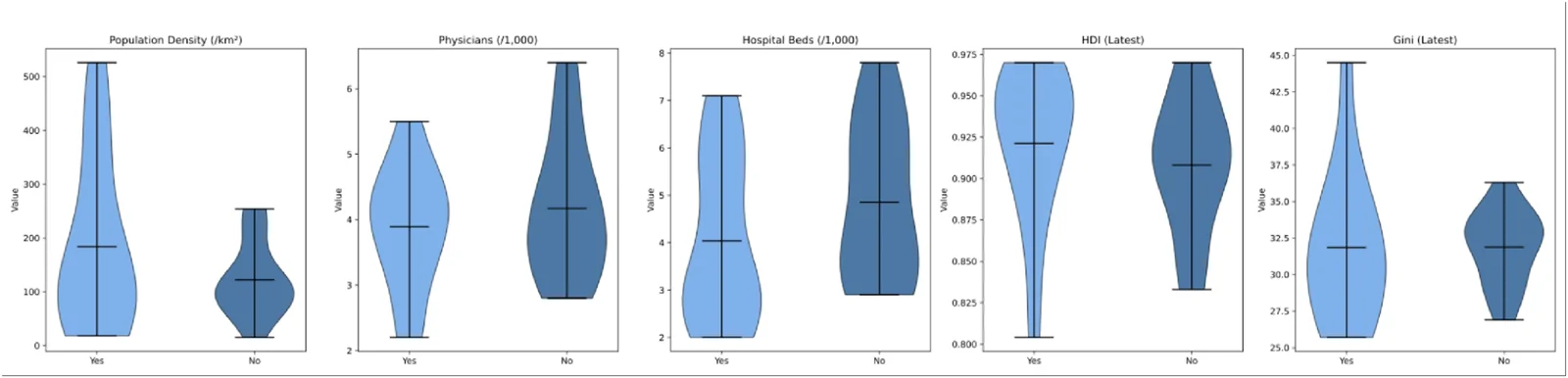
Violin plots showing national indicators according to outpatient ECT reimbursement status: (A) Human Development Index (HDI), (B) Gini coefficient, (C) population density, (D) physicians per 1,000 inhabitants, and (E) hospital beds per 1,000 inhabitants.

**Fig. 4:**
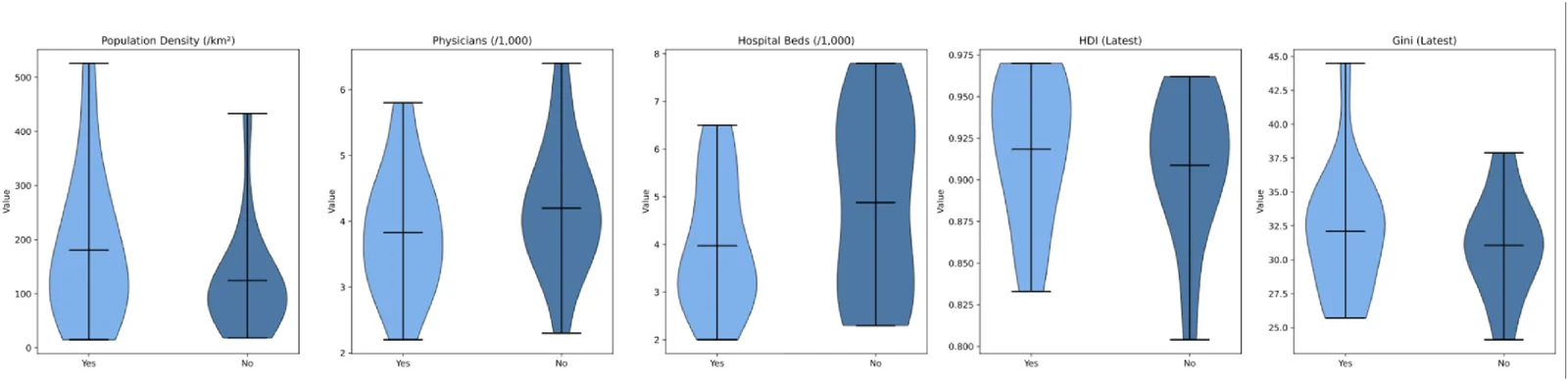
Violin plots showing national indicators according to rTMS reimbursement status: (A) Human Development Index (HDI), (B) Gini coefficient, (C) population density, (D) physicians per 1,000 inhabitants, and (E) hospital beds per 1,000 inhabitants.

## 4 Discussion

In this study, we examined the variation in public health reimbursement policies for ECT and rTMS across 28 European countries. Our analysis revealed substantial disparities in the availability of reimbursement for both treatments, particularly in outpatient settings.

For ECT, while inpatient reimbursement is universally available across all countries surveyed, outpatient reimbursement showed considerable variation, apparently following a geographic pattern. Outpatient ECT reimbursement was more commonly available in Scandinavian and Western European countries, whereas it was frequently absent in Central, Eastern, and Southern Europe. This suggests a regional divergence in health policy approaches to outpatient mental health services, despite consistent evidence supporting the efficacy of ECT across settings in improving clinical outcomes and reducing both personal and societal economic burden (Slade et al., 2017; Ross et al., 2018).

rTMS reimbursement policies demonstrated greater variability and lacked a discernible geographic pattern. Reimbursement for rTMS was limited overall, with high variability between countries that did not align with any obvious regional, economic, or systemic criteria.

Additional analyses that explored whether differences in reimbursement policies could be explained by variation in developmental status, healthcare infrastructure, or population density did not indicate any significant associations. Overall, these findings indicate no strong link between national development or healthcare capacity and reimbursement status. This suggests that the observed variability in reimbursement policies is likely influenced by factors beyond the developmental and healthcare system indicators examined in the present study, potentially including cultural attitudes, clinical traditions, knowledge and awareness of neurostimulation treatments, stigma, and health policy priorities. For rTMS, the absence of any systematic association further underscores the fragmented and inconsistent policy landscape across Europe.

These findings have important policy implications. The striking variability in reimbursement does not correspond to the robust and growing evidence base supporting both ECT and rTMS in the treatment of mental disorders. For ECT, while the widespread availability of inpatient reimbursement is encouraging, the lack of support for outpatient services remains noteworthy. Outpatient ECT can represent a viable option for carefully selected patients who do not require hospitalization. However, its implementation requires specialized infrastructure, anesthesia support, monitoring procedures, and appropriately trained staff. Therefore, the absence of outpatient reimbursement in some countries may partly reflect these practical and organizational challenges. Evidence shows that, even in countries where ECT is fully reimbursed in inpatient and outpatient settings such as the UK, it is used in only 16% of patients with difficult to treat depression open to specialist care (30). This observation suggests that factors beyond reimbursement status, such as availability of specialized services, workforce expertise, treatment traditions, patient preferences, and stigma, may also influence treatment utilization. These factors were not assessed in the present study (Lauber et al., 2005; Loh et al., 2013).

For rTMS, the observed inconsistency in reimbursement is particularly concerning, given the therapy's documented efficacy (lower than ECT's but still higher than many other therapeutic options) and favorable safety profile. At the same time, reimbursement decisions may also be influenced by differences in certification requirements, treatment standards, and national regulatory frameworks that were not assessed in the present study. The unsystematic nature of current reimbursement policies may lead to inequitable access to care and missed opportunities for symptom relief in patients with treatment-resistant conditions.

In light of these findings, we call on European health policy makers and stakeholders to re-evaluate current reimbursement practices. We advocate for policy alignment across Europe based on established scientific evidence and international clinical guidelines to ensure equitable and effective access to ECT and rTMS. Potential next steps may include greater international exchange of best practices, educational initiatives for clinicians and healthcare stakeholders, and evidence-based evaluation of reimbursement frameworks across healthcare systems. A more coordinated, evidence-based approach is essential to improve patient outcomes and reduce disparities in access to mental healthcare across Europe.

Such alignment is particularly crucial for individuals with the most severe forms of mental illness, who are already disproportionately affected by stigma and structural barriers to care. However, reimbursement policies represent only one factor influencing access to ECT and rTMS; infrastructure, availability of specialized services, workforce expertise, institutional treatment practices, and patient-related factors may also contribute. Ensuring equitable access to effective treatments like ECT and rTMS is not only a matter of clinical efficacy, but also of social justice in mental healthcare across Europe (31).

## 5 Limitations

This study has several limitations. First, our analyses were restricted to public reimbursement policies and did not consider private or supplemental insurance coverage. Consequently, overall access to ECT and rTMS may be underestimated in some countries where treatment is available through private healthcare systems. Second, 16 of the 44 countries in the broader European region were excluded because reliable reimbursement information could not be obtained or independently verified; as our study relied on verifiable data sources, we did not systematically collect information on ECT or rTMS access in these countries, and their exclusion may limit the generalizability of our findings. Third, reimbursement status alone does not fully explain treatment utilization: factors such as availability of specialized services, workforce expertise, treatment traditions, patient preferences, and stigma likely also contribute to access and uptake but were not systematically assessed in the present study. Finally, differences in certification requirements, treatment standards, and national regulatory frameworks may further contribute to the observed variability in reimbursement policies and were beyond the scope of this study.

## Contributors

NO and JR planned and conceptualized the study. All authors wrote and edited this manuscript and take joint responsibility for this content.

## Declaration of competing interest

The authors declare that they have no known competing financial interests or personal relationships that could have appeared to influence the work reported in this paper.
